# Supplementary material for: The effect of calcium supplementation in people under 35 years old: A systematic review and meta-analysis of randomized controlled trials
Source: eLife. 2022 Sep 27;11:e79002. doi: 10.7554/eLife.79002 (PMC9514846; doi:10.7554/eLife.79002)
Supplement: Figure 2—source data 1. [file elife-79002-fig2-data1.pdf]

Figure 2 - source data 1. Forest plots for the association between calcium supplementation and the accretion of lumbar spine bone mineral density (LSBMD)

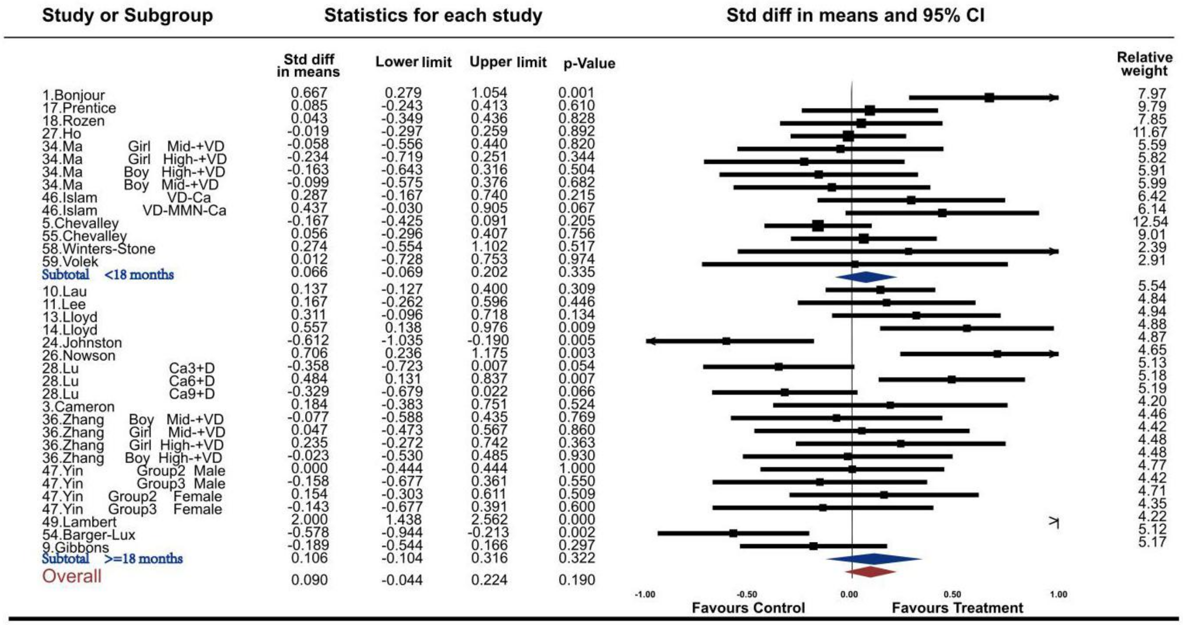

Study or Subgroup	Std diff in means	Lower limit	Upper limit	p-Value
1.Bonjour	0.667	0.279	1.054	0.001
17.Prentice	0.085	-0.243	0.413	0.610
18.Rozen	0.043	-0.349	0.436	0.828
27.Ho	-0.019	-0.297	0.259	0.892
34.Ma Girl Mid-+VD	-0.058	-0.556	0.440	0.820
34.Ma Girl High-+VD	-0.234	-0.719	0.251	0.344
34.Ma Boy High-+VD	-0.163	-0.643	0.316	0.504
34.Ma Boy Mid-+VD	-0.099	-0.575	0.376	0.682
46.Islam VD-Ca	0.287	-0.167	0.740	0.215
46.Islam VD-MMN-Ca	0.437	-0.030	0.905	0.067
5.Chevalley	-0.167	-0.425	0.091	0.205
55.Chevalley	0.056	-0.296	0.407	0.756
58.Winters-Stone	0.274	-0.554	1.102	0.517
59.Volek	0.012	-0.728	0.753	0.974
Subtotal <18 months	0.066	-0.069	0.202	0.335
10.Lau	0.137	-0.127	0.400	0.309
11.Lee	0.167	-0.262	0.596	0.446
13.Lloyd	0.311	-0.096	0.718	0.134
14.Lloyd	0.557	0.138	0.976	0.009
24.Johnston	-0.612	-1.035	-0.190	0.005
26.Nowson	0.706	0.236	1.175	0.003
28.Lu Ca3+D	-0.358	-0.723	0.007	0.054
28.Lu Ca6+D	0.484	0.131	0.837	0.007
28.Lu Ca9+D	-0.329	-0.679	0.022	0.066
3.Cameron	0.184	-0.383	0.751	0.524
36.Zhang Boy Mid-+VD	-0.077	-0.588	0.435	0.769
36.Zhang Girl Mid-+VD	0.047	-0.473	0.567	0.860
36.Zhang Girl High-+VD	0.235	-0.272	0.742	0.363
36.Zhang Boy High-+VD	-0.023	-0.530	0.485	0.930
47.Yin Group2 Male	0.000	-0.444	0.444	1.000
47.Yin Group3 Male	-0.158	-0.677	0.361	0.550
47.Yin Group2 Female	0.154	-0.303	0.611	0.509
47.Yin Group3 Female	-0.143	-0.677	0.391	0.600
49.Lambert	2.000	1.438	2.562	0.000
54.Barger-Lux	-0.578	-0.944	-0.213	0.002
9.Gibbons	-0.189	-0.544	0.166	0.297
Subtotal >=18 months	0.106	-0.104	0.316	0.322
Overall	0.090	-0.044	0.224	0.190
