# Supplementary material for: The effect of calcium supplementation in people under 35 years old: A systematic review and meta-analysis of randomized controlled trials
Source: eLife. 2022 Sep 27;11:e79002. doi: 10.7554/eLife.79002 (PMC9514846; doi:10.7554/eLife.79002)

Figure 2 - source data 2. Forest plots for the association between calcium supplementation and the accretion of femoral neck bone mineral density (FNBMD)

Study or Subgroup	Std diff in means	Lower limit	Upper limit	p-Value	Relative weight
1.Bonjour	2.250	1.768	2.732	0.000	7.15
18.Rozen	0.038	-0.354	0.430	0.848	7.36
34.Ma Girl Mid-+VD	0.034	-0.463	0.532	0.892	7.10
34.Ma Girl High-+VD	0.215	-0.269	0.700	0.384	7.14
34.Ma Boy High-+VD	-0.112	-0.592	0.367	0.646	7.15
34.Ma Boy Mid-+VD	-0.232	-0.709	0.245	0.341	7.16
41.Arab Ca+VD Ex-	2.167	1.198	3.135	0.000	5.68
41.Arab Ca+VD Ex+	3.769	2.535	5.004	0.000	4.85
45.Hemayat* Ca+VD Tr-	1.519	0.524	2.514	0.003	5.59
45.Hemayat* Ca+VD Tr+	1.902	0.846	2.959	0.000	5.40
46.Islam VD-Ca	0.978	0.501	1.455	0.000	7.16
46.Islam VD-MMN-Ca	1.280	0.773	1.787	0.000	7.08
5.Chevalley	0.000	-0.257	0.257	1.000	7.61
55.Chevalley	0.141	-0.211	0.493	0.433	7.44
58.Winters-Stone	0.047	-0.777	0.872	0.911	6.14
Subtotal <18 months	0.824	0.383	1.266	0.000	
10.Lau	0.099	-0.164	0.362	0.460	13.20
11.Lee	-0.056	-0.484	0.373	0.799	11.61
24.Johnston	1.381	0.921	1.841	0.000	11.27
26.Nowson	0.944	0.464	1.425	0.000	11.05
3.Cameron	-0.054	-0.620	0.511	0.850	10.14
36.Zhang Boy Mid-+VD	0.121	-0.391	0.632	0.644	10.72
36.Zhang Girl Mid-+VD	0.209	-0.313	0.730	0.433	10.62
36.Zhang Girl High-+VD	0.716	0.195	1.237	0.007	10.62
36.Zhang Boy High-+VD	0.052	-0.455	0.559	0.841	10.77
Subtotal >=18 months	0.378	0.047	0.709	0.025	
Overall	0.627	0.338	0.915	0.000	

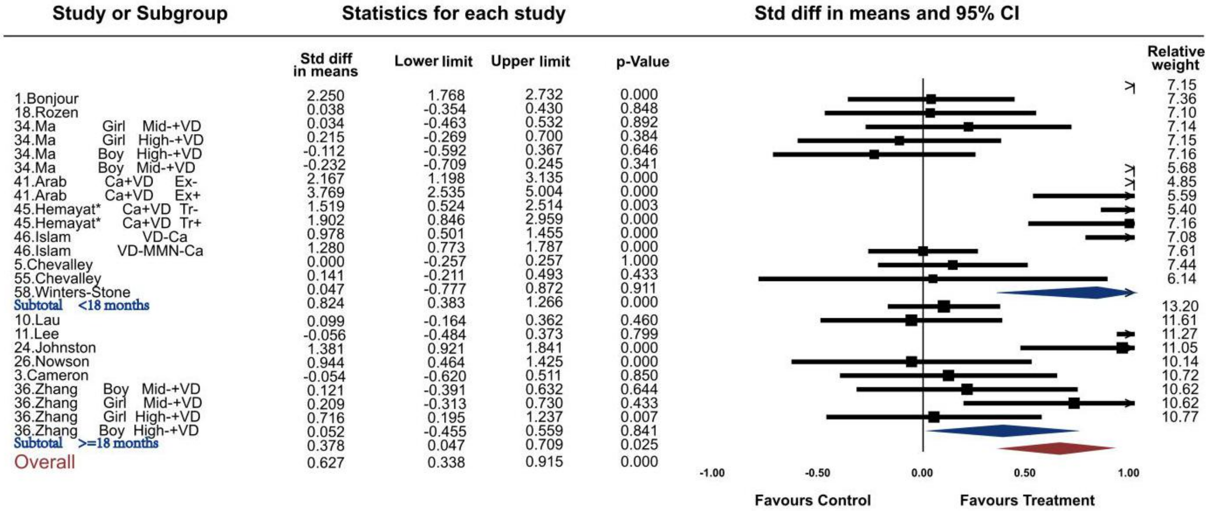

Supplement: Figure 2—source data 2. [file elife-79002-fig2-data2.pdf]
