# Supplementary material for: The effect of calcium supplementation in people under 35 years old: A systematic review and meta-analysis of randomized controlled trials
Source: eLife. 2022 Sep 27;11:e79002. doi: 10.7554/eLife.79002 (PMC9514846; doi:10.7554/eLife.79002)

Figure 2 - source data 3. Forest plots for the association between calcium supplementation and the accretion of total hip bone mineral density (THBMD)

Study or Subgroup	Std diff in means	Lower limit	Upper limit	p-Value	Relative weight
27.Ho	0.085	-0.194	0.363	0.551	41.07
34.Ma Girl Mid-+VD	0.078	-0.420	0.576	0.758	12.82
34.Ma Girl High-+VD	0.200	-0.285	0.685	0.419	13.54
34.Ma Boy High-+VD	0.127	-0.352	0.607	0.603	13.84
34.Ma Boy Mid-+VD	-0.126	-0.602	0.350	0.604	14.05
58.Winters-Stone	0.096	-0.729	0.921	0.819	4.67
Subtotal <18 months	0.076	-0.102	0.255	0.402	
10.Lau	0.102	-0.162	0.365	0.449	8.79
26.Nowson	0.683	0.214	1.151	0.004	8.25
28.Lu Ca3+D	-0.634	-1.005	-0.264	0.001	8.54
28.Lu Ca6+D	0.717	0.358	1.076	0.000	8.57
28.Lu Ca9+D	-0.528	-0.882	-0.174	0.003	8.58
36.Zhang Boy Mid-+VD	0.278	-0.236	0.791	0.289	8.11
36.Zhang Girl Mid-+VD	0.274	-0.248	0.797	0.304	8.08
36.Zhang Girl High-+VD	0.743	0.221	1.265	0.005	8.08
36.Zhang Boy High-+VD	0.108	-0.399	0.616	0.676	8.13
49.Lambert	2.500	1.888	3.112	0.000	7.77
54.Barger-Lux	-0.878	-1.233	-0.524	0.000	8.58
9.Gibbons	1.073	0.695	1.452	0.000	8.52
Subtotal >=18 months	0.351	-0.102	0.805	0.129	
Overall	0.257	-0.053	0.566	0.104	

Std diff in means and 95% CI

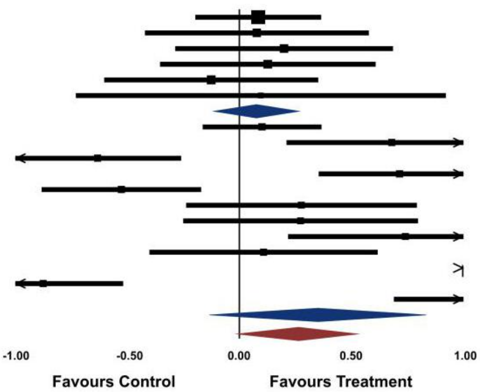

Supplement: Figure 2—source data 3. [file elife-79002-fig2-data3.pdf]
