# Supplementary material for: The effect of calcium supplementation in people under 35 years old: A systematic review and meta-analysis of randomized controlled trials
Source: eLife. 2022 Sep 27;11:e79002. doi: 10.7554/eLife.79002 (PMC9514846; doi:10.7554/eLife.79002)

Figure 2 - source data 4. Forest plots for the association between calcium supplementation and the accretion of total body bone mineral density (TBBMD)

Study or Subgroup	Std diff in means	Lower limit	Upper limit	p-Value	Relative weight
17.Prentice	0.099	-0.229	0.428	0.552	9.45
18.Rozen	0.109	-0.283	0.502	0.585	8.98
25.Molgaard A group	0.388	-0.123	0.899	0.136	8.06
25.Molgaard B group	0.087	-0.463	0.637	0.756	7.74
34.Ma Girl Mid-+VD	-0.100	-0.598	0.398	0.694	8.16
34.Ma Girl High-+VD	-0.145	-0.629	0.339	0.556	8.27
34.Ma Boy High-+VD	0.295	-0.187	0.776	0.231	8.29
34.Ma Boy Mid-+VD	0.149	-0.327	0.625	0.539	8.33
40.Khadilkar Ca	1.236	0.873	1.599	0.000	9.20
40.Khadilkar Ca+MZ	1.123	0.767	1.480	0.000	9.25
42.Ekbote	0.400	-0.120	0.920	0.132	7.98
59.Volek	0.017	-0.724	0.758	0.964	6.31
Subtotal <18 months	0.324	0.035	0.614	0.028	
10.Lau	0.101	-0.162	0.364	0.453	4.25
13.Lloyd	0.328	-0.079	0.735	0.115	3.87
14.Lloyd	0.613	0.193	1.034	0.004	3.83
15.Matkovic 4y	0.192	-0.073	0.457	0.155	4.25
15.Matkovic 7y	0.108	-0.189	0.405	0.475	4.17
2.Cadogan	0.401	-0.037	0.839	0.073	3.78
28.Lu Ca3+D	-1.119	-1.507	-0.730	0.000	3.93
28.Lu Ca6+D	0.696	0.337	1.054	0.000	4.01
28.Lu Ca9+D	-0.126	-0.474	0.223	0.479	4.04
36.Zhang Boy Mid-+VD	0.133	-0.379	0.645	0.610	3.56
36.Zhang Girl Mid-+VD	-0.382	-0.906	0.143	0.154	3.52
36.Zhang Girl High-+VD	-0.106	-0.612	0.400	0.681	3.58
36.Zhang Boy High-+VD	0.073	-0.434	0.581	0.776	3.57
4.Cheng Ca	0.076	-0.363	0.515	0.734	3.78
4.Cheng Ca+D	0.080	-0.347	0.507	0.713	3.82
4.Cheng Cheese as daily supply	0.132	-0.312	0.577	0.559	3.76
47.Yin Group2 Male	0.390	-0.058	0.838	0.088	3.75
47.Yin Group3 Male	0.250	-0.270	0.770	0.346	3.53
47.Yin Group2 Female	0.333	-0.126	0.793	0.155	3.72
47.Yin Group3 Female	0.333	-0.203	0.870	0.224	3.48
49.Lambert	3.760	2.997	4.524	0.000	2.80
50.Zhu Ca	0.462	0.202	0.723	0.001	4.26
50.Zhu CaD	0.902	0.633	1.170	0.000	4.24
7.Du M+Ca	0.397	0.137	0.656	0.003	4.26
7.Du M+Ca+D	0.676	0.413	0.939	0.000	4.25
9.Gibbons	0.733	0.367	1.099	0.000	3.99
Subtotal >=18 months	0.334	0.129	0.539	0.001	
Overall	0.330	0.163	0.496	0.000	

Std diff in means and 95% CI

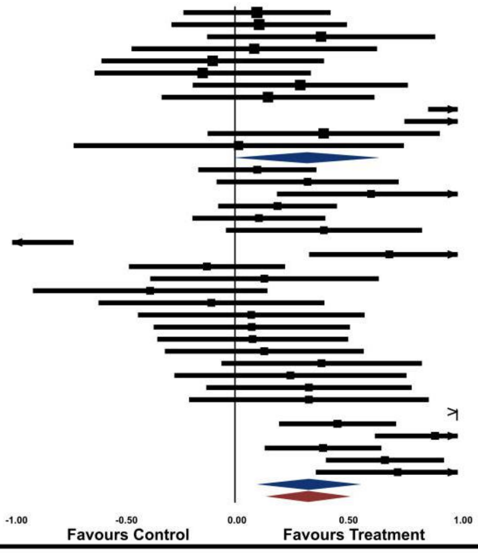

Supplement: Figure 2—source data 4. [file elife-79002-fig2-data4.pdf]
