# Supplementary material for: The effect of calcium supplementation in people under 35 years old: A systematic review and meta-analysis of randomized controlled trials
Source: eLife. 2022 Sep 27;11:e79002. doi: 10.7554/eLife.79002 (PMC9514846; doi:10.7554/eLife.79002)

Figure 3 - source data 1. Forest plots for the association between calcium supplementation and the accretion of lumbar spine bone mineral content (LSBMC)

Study or Subgroup		Std diff in means	Lower limit	Upper limit	p-Value	Relative weight
17.Prentice		0.045	-0.283	0.373	0.788	8.50
18.Rozen		0.188	-0.205	0.581	0.347	6.81
20.Stear	exercise	0.013	-0.440	0.466	0.955	5.61
20.Stear	non-exercise	0.102	-0.422	0.626	0.703	4.51
22.Courteix	exercise	0.749	0.093	1.406	0.025	3.13
22.Courteix	sedentary	0.078	-0.675	0.832	0.838	2.47
23.Sandra	exercise	0.645	-0.046	1.335	0.067	2.87
23.Sandra	non-exercise	-1.000	-1.740	-0.260	0.008	2.55
27.Ho		0.000	-0.278	0.278	1.000	10.12
34.Ma	Girl Mid-+VD	-0.346	-0.848	0.155	0.176	4.82
34.Ma	Girl High-+VD	-0.181	-0.665	0.303	0.464	5.08
34.Ma	Boy High-+VD	-0.061	-0.540	0.418	0.804	5.16
34.Ma	Boy Mid-+VD	-0.185	-0.661	0.292	0.448	5.21
37.Ward		0.000	-0.438	0.438	1.000	5.87
46.Islam	VD-Ca	0.201	-0.251	0.653	0.384	5.62
46.Islam	VD-MMN-Ca	0.268	-0.196	0.732	0.258	5.41
51.Ward	GYMNASTS	0.220	-0.410	0.850	0.493	3.36
51.Ward	NORMAL	0.000	-0.653	0.653	1.000	3.16
53.Bass	exercise	0.781	0.145	1.416	0.016	3.31
53.Bass	Nonexercise	0.000	-0.575	0.575	1.000	3.89
59.Volek		0.095	-0.646	0.836	0.802	2.54
Subtotal	<18 months	0.063	-0.063	0.190	0.328	
10.Lau		0.152	-0.111	0.415	0.258	7.45
11.Lee		0.298	-0.132	0.729	0.175	6.83
13.Lloyd		0.189	-0.217	0.594	0.361	6.94
14.Lloyd		0.510	0.092	0.928	0.017	6.89
31.Vogel		0.934	0.524	1.344	0.000	6.92
36.Zhang	Boy Mid-+VD	-0.143	-0.655	0.369	0.584	6.48
36.Zhang	Girl Mid-+VD	-0.101	-0.621	0.419	0.703	6.44
36.Zhang	Girl High-+VD	0.200	-0.306	0.707	0.438	6.50
36.Zhang	Boy High-+VD	-0.344	-0.855	0.166	0.186	6.48
47.Yin	Group2 Male	0.236	-0.210	0.681	0.300	6.77
47.Yin	Group3 Male	0.062	-0.456	0.580	0.815	6.45
47.Yin	Group2 Female	0.087	-0.370	0.543	0.710	6.72
47.Yin	Group3 Female	-0.209	-0.744	0.326	0.444	6.37
49.Lambert		3.167	2.478	3.855	0.000	5.66
54.Barger-Lux		-0.247	-0.607	0.113	0.179	7.12
Subtotal	>=18 months	0.293	-0.015	0.602	0.062	
Overall		0.163	0.008	0.317	0.039	

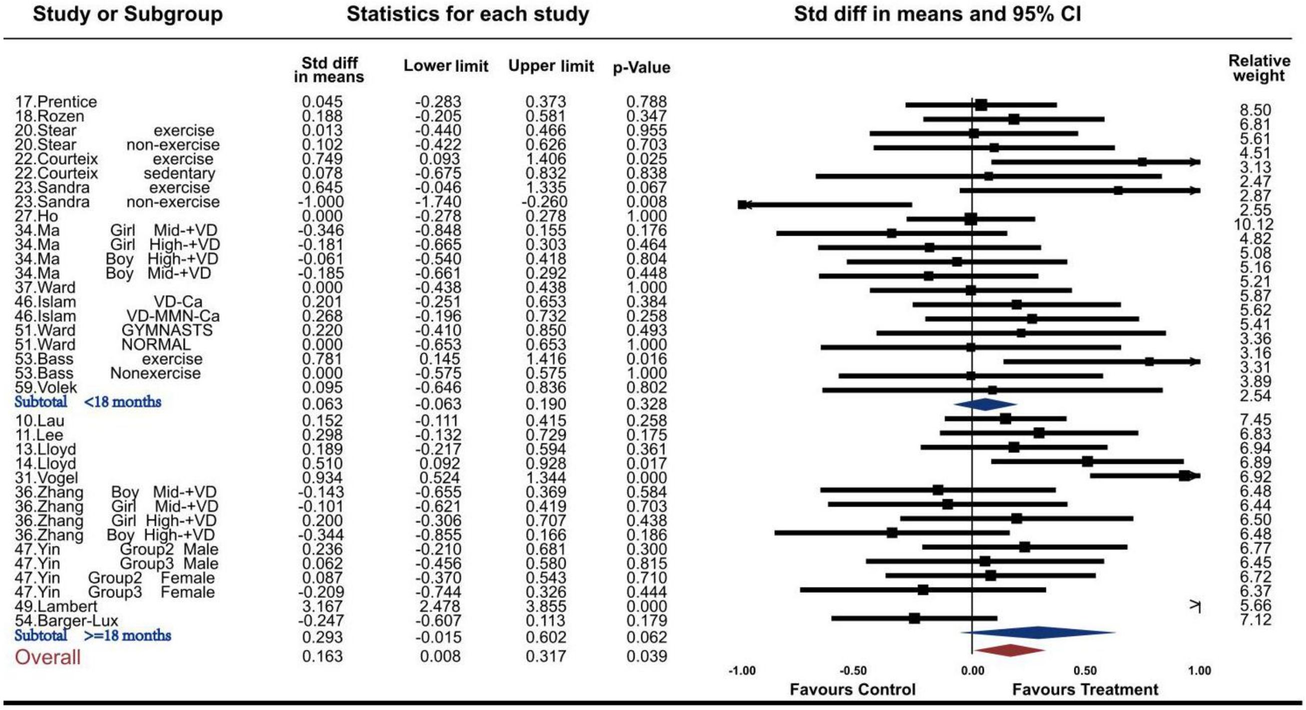

Supplement: Figure 3—source data 1. [file elife-79002-fig3-data1.pdf]
