# Supplementary material for: The effect of calcium supplementation in people under 35 years old: A systematic review and meta-analysis of randomized controlled trials
Source: eLife. 2022 Sep 27;11:e79002. doi: 10.7554/eLife.79002 (PMC9514846; doi:10.7554/eLife.79002)

Figure 3 - source data 2. Forest plots for the association between calcium supplementation and the accretion of femoral neck bone mineral content (FNBMC)

Study or Subgroup	Std diff in means	Lower limit	Upper limit	p-Value	Relative weight
18.Rozen	0.090	-0.303	0.482	0.654	12.60
22.Courteix exercise	1.098	0.424	1.773	0.001	9.56
22.Courteix sedentary	0.467	-0.295	1.229	0.229	8.69
34.Ma Girl Mid-+VD	-0.346	-0.847	0.156	0.176	11.42
34.Ma Girl High-+VD	0.241	-0.244	0.727	0.329	11.60
34.Ma Boy High-+VD	0.897	0.394	1.399	0.000	11.41
34.Ma Boy Mid-+VD	0.668	0.179	1.156	0.007	11.57
46.Islam VD-Ca	0.890	0.418	1.363	0.000	11.74
46.Islam VD-MMN-Ca	1.221	0.717	1.724	0.000	11.40
Subtotal <18 months	0.569	0.223	0.914	0.001	
10.Lau	0.172	-0.091	0.436	0.200	41.11
11.Lee	0.009	-0.420	0.437	0.968	15.56
36.Zhang Boy Mid-+VD	-0.058	-0.570	0.453	0.823	10.92
36.Zhang Girl Mid-+VD	0.054	-0.466	0.574	0.840	10.55
36.Zhang Girl High-+VD	0.519	0.005	1.032	0.048	10.82
36.Zhang Boy High-+VD	-0.183	-0.691	0.326	0.481	11.04
Subtotal >=18 months	0.107	-0.062	0.276	0.213	
Overall	0.364	0.134	0.595	0.002	

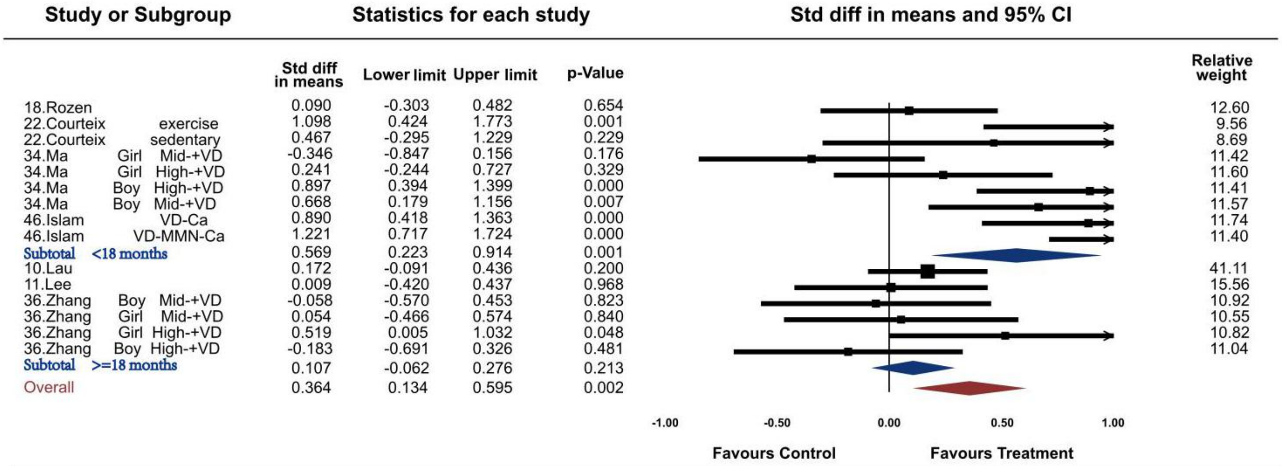

Supplement: Figure 3—source data 2. [file elife-79002-fig3-data2.pdf]
