# Supplementary material for: The effect of calcium supplementation in people under 35 years old: A systematic review and meta-analysis of randomized controlled trials
Source: eLife. 2022 Sep 27;11:e79002. doi: 10.7554/eLife.79002 (PMC9514846; doi:10.7554/eLife.79002)

Figure 3 - source data 3. Forest plots for the association between calcium supplementation and the accretion of total hip bone mineral content (THBMC)

Study or Subgroup	Std diff in means	Lower limit	Upper limit	p-Value	Relative weight
27.Ho	-0.070	-0.348	0.209	0.624	21.96
34.Ma Girl Mid-+VD	-0.117	-0.616	0.381	0.644	15.10
34.Ma Girl High-+VD	-0.111	-0.595	0.373	0.653	15.50
34.Ma Boy High-+VD	0.059	-0.420	0.538	0.810	15.64
34.Ma Boy Mid-+VD	-0.176	-0.652	0.300	0.468	15.72
37.Ward	-0.981	-1.444	-0.517	0.000	16.07
Subtotal <18 months	-0.226	-0.514	0.061	0.123	
10.Lau	0.168	-0.096	0.431	0.212	12.84
31.Vogel	2.820	2.271	3.369	0.000	12.36
36.Zhang Boy Mid-+VD	0.095	-0.416	0.607	0.715	12.44
36.Zhang Girl Mid-+VD	0.198	-0.323	0.719	0.457	12.42
36.Zhang Girl High-+VD	0.472	-0.040	0.984	0.071	12.44
36.Zhang Boy High-+VD	-0.100	-0.607	0.408	0.700	12.45
49.Lambert	1.417	0.904	1.930	0.000	12.44
54.Barger-Lux	-1.936	-2.346	-1.525	0.000	12.63
Subtotal >=18 months	0.385	-0.495	1.264	0.392	
Overall	0.116	-0.382	0.614	0.648	

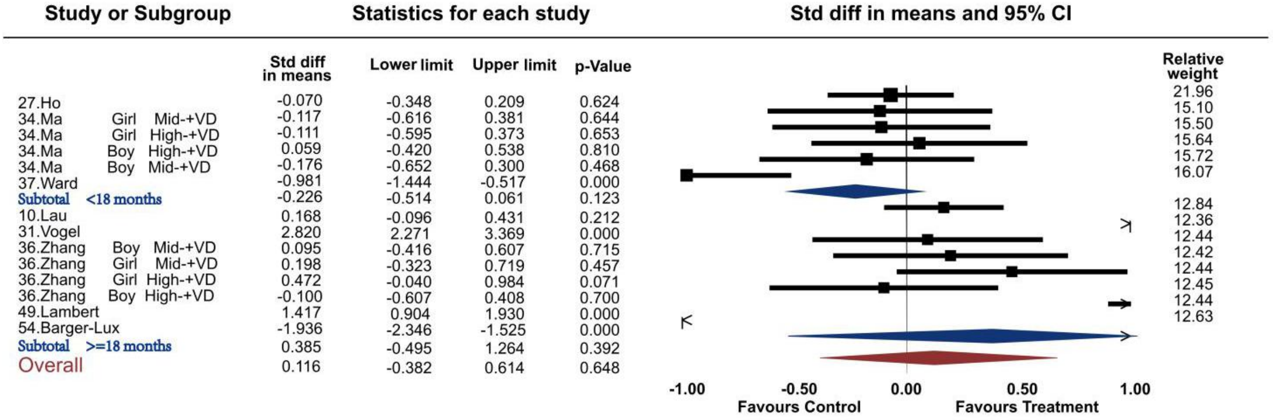

Supplement: Figure 3—source data 3. [file elife-79002-fig3-data3.pdf]
