# Supplementary material for: The effect of calcium supplementation in people under 35 years old: A systematic review and meta-analysis of randomized controlled trials
Source: eLife. 2022 Sep 27;11:e79002. doi: 10.7554/eLife.79002 (PMC9514846; doi:10.7554/eLife.79002)

Figure 3 - source data 4. Forest plots for the association between calcium supplementation and the accretion of total body bone mineral content (TBBMC)

Study or Subgroup	Std diff in means	Lower limit	Upper limit	p-Value	Relative weight
16.Moyer-Mileur Ca+D	0.028	-0.437	0.493	0.906	4.04
17.Prentice	0.077	-0.251	0.405	0.643	4.37
18.Rozen	-0.011	-0.403	0.381	0.957	4.22
19.Specker fine motor	0.333	-0.083	0.749	0.116	4.16
19.Specker gross motor	0.667	0.237	1.096	0.002	4.13
20.Stear exercise	0.037	-0.416	0.489	0.874	4.07
20.Stear non-exercise	0.061	-0.463	0.585	0.820	3.88
22.Courteix exercise	0.878	0.215	1.540	0.009	3.50
22.Courteix sedentary	-0.156	-0.911	0.598	0.684	3.25
23.Sandra exercise	-1.065	-1.785	-0.346	0.004	3.34
23.Sandra non-exercise	-0.257	-0.958	0.445	0.473	3.39
25.Molgaard A group	0.008	-0.498	0.514	0.975	3.93
25.Molgaard B group	-0.243	-0.795	0.309	0.388	3.81
34.Ma Girl Mid-+VD	0.150	-0.349	0.648	0.556	3.95
34.Ma Girl High-+VD	0.107	-0.376	0.591	0.663	3.99
34.Ma Boy High-+VD	0.075	-0.404	0.554	0.759	4.00
34.Ma Boy Mid-+VD	-0.154	-0.630	0.322	0.527	4.01
37.Ward	-0.827	-1.283	-0.370	0.000	4.06
40.Khadilkar Ca	1.373	1.004	1.743	0.000	4.28
40.Khadilkar Ca+MZ	1.129	0.772	1.485	0.000	4.31
42.Ekbote	0.616	0.089	1.144	0.022	3.87
51.Ward GYMNASTS	0.436	-0.199	1.072	0.178	3.57
51.Ward NORMAL	0.000	-0.653	0.653	1.000	3.52
53.Bass exercise	1.368	0.688	2.049	0.000	3.45
53.Bass Nonexercise	-1.213	-1.838	-0.588	0.000	3.60
59.Volek	0.019	-0.722	0.760	0.960	3.28
Subtotal <18 months	0.146	-0.095	0.387	0.235	
10.Lau	0.140	-0.123	0.404	0.296	4.74
12.Lee	0.201	-0.108	0.510	0.203	4.54
13.Lloyd	0.135	-0.270	0.539	0.515	4.10
14.Lloyd	0.540	0.122	0.959	0.011	4.04
2.Cadogan	0.380	-0.057	0.818	0.089	3.95
28.Lu Ca3+D	-0.586	-0.955	-0.216	0.002	4.27
28.Lu Ca6+D	0.202	-0.147	0.551	0.257	4.36
28.Lu Ca9+D	0.079	-0.269	0.428	0.656	4.37
3.Cameron	0.219	-0.349	0.786	0.450	3.35
31.Vogel	0.455	0.061	0.850	0.024	4.15
36.Zhang Boy Mid-+VD	0.110	-0.402	0.621	0.674	3.61
36.Zhang Girl Mid-+VD	-0.267	-0.789	0.255	0.316	3.56
36.Zhang Girl High-+VD	-0.479	-0.992	0.033	0.067	3.60
36.Zhang Boy High-+VD	-0.269	-0.779	0.240	0.300	3.61
4.Cheng Ca	0.063	-0.375	0.502	0.778	3.95
4.Cheng Ca+D	-0.084	-0.511	0.343	0.700	4.00
4.Cheng Cheese	0.112	-0.332	0.556	0.620	3.92
47.Yin Group2 Male	0.609	0.155	1.063	0.009	3.87
47.Yin Group3 Male	0.329	-0.192	0.850	0.216	3.56
47.Yin Group2 Female	0.046	-0.411	0.502	0.844	3.86
47.Yin Group3 Female	0.112	-0.422	0.645	0.681	3.50
49.Lambert	2.214	1.632	2.797	0.000	3.29
54.Barger-Lux	-0.716	-1.086	-0.347	0.000	4.27
7.Du M+Ca	0.153	-0.104	0.411	0.243	4.76
7.Du M+Ca+D	0.206	-0.050	0.463	0.115	4.76
Subtotal >=18 months	0.143	-0.027	0.313	0.100	
Overall	0.149	0.006	0.291	0.040	

Std diff in means and 95% CI

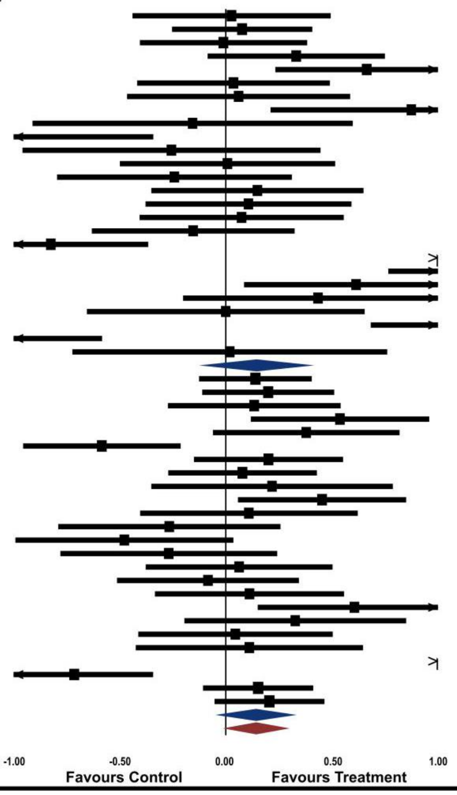

Supplement: Figure 3—source data 4. [file elife-79002-fig3-data4.pdf]
