# Supplementary material for: The effect of calcium supplementation in people under 35 years old: A systematic review and meta-analysis of randomized controlled trials
Source: eLife. 2022 Sep 27;11:e79002. doi: 10.7554/eLife.79002 (PMC9514846; doi:10.7554/eLife.79002)
Supplement: Supplementary file 1. [file elife-79002-supp1.docx]

**Supplementary file 1**: Search strategies

*Pubmed*

#1. **(((((((((((calcium[MeSH Terms]) OR (calcium[Title/Abstract])) OR (calcium carbonate[Title/Abstract])) OR (calcium citrate[Title/Abstract])) OR (calcium pills[Title/Abstract])) OR (calcium supplement[Title/Abstract])) OR (Ca2[Title/Abstract])) OR (dairy product[Title/Abstract])) OR (milk[Title/Abstract])) OR (yogurt[Title/Abstract])) OR (cheese[Title/Abstract])) OR (dietary supplement[Title/Abstract])**

#2. **(((randomized controlled trial[Publication Type]) OR (randomized controlled trial[Title/Abstract])) OR (clinical trials[Title/Abstract])) OR (RCT[Title/Abstract])**

#3. #1 AND #2

#4. **(((((Bone Density[MeSH Terms]) OR (bone density[Title/Abstract])) OR (bone mineral density[Title/Abstract])) OR (bone mineral densities[Title/Abstract])) OR (bone mineral content[Title/Abstract])) OR (bone mineral contents[Title/Abstract])**

#5. #3 AND #4

*EMBASE*

#1. ’**calcium’**:ti,ab,kw OR *’***calcium carbonate’**:ti,ab,kw OR ’**calcium citrate’**:ti,ab,kw OR ’**calcium pills’**:ti,ab,kw OR *’***calcium supplement’**:ti,ab,kw OR *’***Ca2’**:ti,ab,kw OR *’***dairy product’**:ti,ab,kw OR *’***milk’**:ti,ab,kw OR *’***yogurt’**:ti,ab,kw OR *’***cheese’**:ti,ab,kw OR *’***dietary supplement’**:ti,ab,kw

#2. *’***randomized controlled trial’**:ti,ab,kw OR *’***clinical trials’**:ti,ab,kw OR ’ RCT **’**:ti,ab,kw

#3. #1 AND #2

#4. ’**bone density’**:ti,ab,kw OR *’***bone mineral density’**:ti,ab,kw OR *’***bone mineral densities’**:ti,ab,kw OR *’***bone mineral content’**:ti,ab,kw OR *’***bone mineral contents’**:ti,ab,kw

#5. #3 AND #4

*ProQuest*

#1. mesh(**calcium) OR mainsubject(calcium) OR ab(calcium carbonate) OR ab(calcium citrate) OR ab(calcium pills) OR ab(calcium supplement) OR ab(Ca2) OR ab(dairy product) OR ab(milk) OR ab(yogurt) OR ab(cheese) OR ab(dietary supplement)**

**#2. mesh(Randomized Controlled Trials as Topic)** OR mesh(controlled clinical trials as topic) OR ab(Randomized Controlled Trial) OR ab(controlled clinical trial) OR ab(controlled trial) OR ab(clinical trial) OR ab(RCT)

#3. #1 AND #2

**#4 mesh(Bone Density) OR mainsubject(bone density) OR ab(bone mineral density) OR ab(bone mineral densities) OR ab(bone mineral content) OR ab(bone mineral contents)**

#5. #3 AND #4

*CENTRAL (Cochrane Central Register of Controlled Trials)*

#1. MeSH descriptor: [calcium] explode all trees

#2. (**calcium carbonate)**:ti,ab,kw OR (**calcium citrate)**:ti,ab,kw OR (**calcium pills)**:ti,ab,kw OR (**calcium supplement)**:ti,ab,kw OR (**Ca2)**:ti,ab,kw OR (**dairy product)**:ti,ab,kw OR (**milk)**:ti,ab,kw OR (**yogurt)**:ti,ab,kw OR (**cheese)**:ti,ab,kw OR (**dietary supplement)**:ti,ab,kw

#3. #1 OR #2

#4. MeSH descriptor: [**Bone Density] explode all trees**

**#5. (bone density)**:ti,ab,kw OR (**bone mineral density)**:ti,ab,kw OR (**bone mineral densities)**:ti,ab,kw OR (**one mineral content)**:ti,ab,kw OR (**bone mineral contents)**:ti,ab,kw

#6. #4 OR #5

#7. #3 AND #6

*WHO Global Index Medicus*

(tw:(calcium)) OR (mj:(calcium)) OR (tw:(calcium carbonate)) OR (tw:(calcium citrate)) OR (tw:(calcium pills)) OR (tw:(calcium supplement)) OR (tw:(Ca2)) OR (tw:(dairy product)) OR (tw:(milk)) OR (tw:(yogurt)) OR (tw:(cheese)) OR (tw:(dietary supplement)) AND (tw:(bone density)) OR (tw:(bone mineral density)) OR (tw:(bone mineral content))

*ClinicalTrials.gov*

Calcium OR calcium supplementation OR milk OR dairy product OR yogurt OR cheese

Applied Filters: Interventional (clinical trial); Child (birth–17); Adult (18–64)

*WHO ICTRP*

Calcium OR milk OR dairy OR yogurt OR cheese in the Intervention

*Wanfang Data (Chinese database)*

主题:("钙") or 题名或关键词:(钙 or 碳酸钙 or 乳酸钙 or 钙片 or 钙补充 or 乳制品 or 牛奶 or 酸奶 or 奶酪 ) and 题名或关键词:(随机对照试验) and 题名或关键词:(骨密度 or 骨矿物密度 or 骨矿物含量)

*China National Knowledge Infrastructure (CNKI)*

[(主题=钙) OR (篇关摘%钙 + 碳酸钙 + 乳酸钙 + 钙片 + 钙补充 + 乳制品 + 牛奶 + 酸奶 + 奶酪) AND (篇关摘%随机对照试验) AND (篇关摘%骨密度 + 骨矿物密度](https://kns.cnki.net/kns8/AdvSearch?id=12&dbcode=CFLS&searchtype=gradeSearch&ishistory=1" \o "(主题=钙) OR (篇关摘%钙 + 碳酸钙 + 乳酸钙 + 钙片 + 钙补充 + 乳制品 + 牛奶 + 酸奶 + 奶酪) AND (篇关摘%随机对照试验) AND (篇关摘%骨密度 + 骨矿物密度 + 骨矿物含量)" \t "https://kns.cnki.net/kns8/manage/_blank)+ 骨矿物含量）
