# Supplementary material for: The effect of calcium supplementation in people under 35 years old: A systematic review and meta-analysis of randomized controlled trials
Source: eLife. 2022 Sep 27;11:e79002. doi: 10.7554/eLife.79002 (PMC9514846; doi:10.7554/eLife.79002)
Supplement: Supplementary file 6. — (A) Cumulative meta-analysis according to sample size in lumbar spine bone mineral density (LSBMD). (B) Cumulative meta-analysis according to sample size in femoral neck bone mineral density (FNBMD). (C) Cumulative meta-analysis according to sample size in total hip bone mineral density (THBMD). (D) Cumulative meta-analysis according to sample size in total body bone mineral density (TBBMD). (E) Cumulative meta-analysis according to sample size in lumbar spine bone mineral content (LSBMC). (F) Cumulative meta-analysis according to sample size in femoral neck bone mineral content (FNBMC). (G) Cumulative meta-analysis according to sample size in total hip bone mineral content (THBMC). (H) Cumulative meta-analysis according to sample size in total body bone mineral content (TBBMC). [file elife-79002-supp6.doc]

**Supplementary file 6A. Cumulative meta-analysis according to sample size in lumbar spine bone mineral density (LSBMD)**

**Supplementary file 6B. Cumulative meta-analysis according to sample size in femoral neck bone mineral density (FNBMD)**

**Supplementary file 6C. Cumulative meta-analysis according to sample size in total hip bone mineral density (THBMD)**

**Supplementary file 6D. Cumulative meta-analysis according to sample size in total body bone mineral density (TBBMD)**

**Supplementary file 6E. Cumulative meta-analysis according to sample size in lumbar spine bone mineral content (LSBMC)**

**Supplementary file 6F. Cumulative meta-analysis according to sample size in femoral neck bone mineral content (FNBMC)**

**Supplementary file 6G. Cumulative meta-analysis according to sample size in total hip bone mineral content (THBMC)**

**Supplementary file 6H. Cumulative meta-analysis according to sample size in total body bone mineral content (TBBMC)**
