# Supplementary material for: The effect of calcium supplementation in people under 35 years old: A systematic review and meta-analysis of randomized controlled trials
Source: eLife. 2022 Sep 27;11:e79002. doi: 10.7554/eLife.79002 (PMC9514846; doi:10.7554/eLife.79002)
Supplement: Supplementary file 7. — (A) Sensitivity analyses by comparisons of the pooled results of the trials included in previous study and trials newly added in our current study of bone mineral density (BMD). (B) Sensitivity analyses by comparisons of the pooled results of the trials included in previous study and trials newly added in our current study of bone mineral content (BMC). [file elife-79002-supp7.doc]

**Supplementary file 7A. Sensitivity analyses by comparisons of the pooled results of the trials included in previous study and trials newly-added in our current study of bone mineral density (BMD)***

| **Subgroups** | **No. of studies or subgroups** | **Effect estimate (95%CI)** | **P-value** | **Heterogeneity between studies** | | **P-value for heterogeneity between subgroups** |
| --- | --- | --- | --- | --- | --- | --- |
|  |  |  |  | **I² (%)** | **P-value** |  |
| **Lumbar Spine** |  | | | | |  |
| Trials included in previous study | 9 | 0.143 (-0.113 to 0.339) | 0.274 | 74.12 | <.001 | 0.673 |
| Trials newly included | 26 | 0.071 (-0.091 to 0.232) | 0.39 | 72.06 | <.001 |  |
| **Femoral Neck** |  | | | | |  |
| Trials included in previous study | 7 | 0.637 (0.003 to 1.271) | 0.049 | 93.86 | <.001 | 0.313 |
| Trials newly included | 17 | 0.603 (0.286 to 0.92) | <.001 | 83.49 | <.001 |  |
| **Total Hip** |  | | | | |  |
| Trials included in previous study | 1 | 0.683 (0.214 to 1.151) | 0.004 | 0.00 | 1.000 | 0.014 |
| Trials newly included | 17 | 0.232 (-0.089 to 0.553) | 0.157 | 89.98 | <.001 |  |
| **Total Body** |  | | | | | |
| Trials included in previous study | 7 | 0.171 (0.036 to 0.307) | 0.013 | 0.00 | 0.935 | 0.507 |
| Trials newly included | 31 | 0.365 (0.161 to 0.569) | <.001 | 87.49 | <.001 |  |

*The previous study are mentioned in the discussion part of our manuscript and are from Winzenberg, T et al.

**Supplementary file 7B. Sensitivity analyses by comparisons of the pooled results of the trials included in previous study and trials newly-added in our current study of bone mineral content (BMC)***

| **Subgroups** | **No. of studies or subgroups** | **Effect estimate (95%CI)** | **P-value** | | | **Heterogeneity between studies** | | **P-value for heterogeneity between subgroups** |
| --- | --- | --- | --- | --- | --- | --- | --- | --- |
|  |  |  |  |  |  | **I² (%)** | **P-value** |  |
| **Lumbar Spine** |  |  | |  |  |  |  |  |
| Trials included in previous study | 10 | 0.144 (-0.065 to 0.353) | 0.177 | | | 42.93 | 0.072 | 0.773 |
| Trials newly included | 26 | 0.174 (-0.024 to 0.373) | 0.086 | | | 78.69 | <.001 |  |
| **Femoral Neck** |  |  | |  |  |  |  |  |
| Trials included in previous study | 4 | 0.348 (-0.097 to 0.792) | 0.125 | | | 63.67 | 0.041 | 0.930 |
| Trials newly included | 11 | 0.369 (0.088 to 0.649) | 0.010 | | | 75.21 | <.001 |  |
| **Total Hip** |  |  | |  |  |  |  |  |
| Trials included in previous study | -- | -- | -- | | | -- | -- | -- |
| Trials newly included | 14 | 0.116 (-0.382 to 0.614) | 0.648 | | | 94.59 | <.001 |  |
| **Total Body** |  |  | |  |  |  |  |  |
| Trials included in previous study | 15 | 0.100 (-0.073 to 0.273) | 0.259 | | | 48.558 | 0.018 | 0.994 |
| Trials newly included | 36 | 0.177 (-0.009 to 0.363) | 0.063 | | | 84.986 | <.001 |  |

*The previous study are mentioned in the discussion part of our manuscript and are from Winzenberg, T et al.
